# Coupling Effects of Straw Return and Fertilization Regime on the Photosynthesis-Soil-Yield Continuum of Spring Maize in Cold Regions

**DOI:** 10.3390/plants15111665

**Published:** 2026-05-29

**Authors:** Wenhui Wang, Bing Yang, Xianghai Meng, Baicheng Wang, Xingzhe Zhang, Ruiyang Sun, Xinrui Shi, Dehai Xu, Xiaoyu Hao

**Affiliations:** 1 Mudanjiang Branch, Heilongjiang Academy of Agricultural Sciences, Mudanjiang 157000, China; 2 Heilongjiang Academy of Black Soil Conservation & Utilization, Harbin 150086, China

**Keywords:** straw return, photosynthetic characteristics, soil nutrients, spring maize, cold regions

## Abstract

Long-term straw return combined with optimized fertilization represents a effective strategy to enhance soil quality and crop productivity in cold regions, yet its integrated effects on the photosynthesis–soil–yield continuum of spring maize remain unclear, particularly under conditions of low accumulated temperature and slow straw decomposition. Based on a 9-year field experiment (2017–2025) conducted in the cold spring maize zone of Northeast China, this study investigated five treatments: CK, CF, CK + S, CF + S, and OPT + S. Photosynthetic parameters at four growth stages, soil nutrients, and grain yield were systematically measured. The results showed that OPT + S achieved the highest grain yield (15,016.11 kg·ha^−1^ in 2025) and maintained superior photosynthetic performance throughout the growing season, with a photosynthetic decline rate of only 37.21% during the grain-filling stage–significantly lower than that of CK (48.20%). Soil available phosphorus (AP) and available potassium (AK) were significantly increased under straw return treatments (CK + S, CF + S, OPT + S). Correlation and stepwise regression analyses identified AP and net photosynthetic rate at the jointing stage (PnJ) as the key drivers of yield, jointly explaining 84% of yield variation. These findings demonstrate that the OPT + S treatment optimizes the coupling among early-stage photosynthesis, soil nutrient availability, and grain yield, providing a practical and high-yielding nutrient management strategy for spring maize in cold regions.

## 1. Introduction

Maize (*Zea mays* L.) is a strategically important crop that plays vital roles in food security, feed production, economic development, and energy supply worldwide. In 2025, China’s maize sowing area and production were 44.96 million ha and 301.24 million t, respectively, ranking among the highest in the world [1]. Such a large-scale cultivation system generates massive amounts of maize straw residues. Improper disposal, such as open burning and random stacking, not only poses severe ecological risks but also causes considerable waste of valuable resources [2,3,4,5,6]. Maize straw is rich in nitrogen, phosphorus, potassium, and other essential nutrients, making it a high-value agricultural resource [7]. As a core practice of conservation agriculture, straw return releases nutrients from crop residues into the soil through decomposition, which effectively improves soil fertility, optimizes soil structure, and reduces the dependence on chemical fertilizers [8,9]. This sustainable management measure has been widely adopted in more than 70 countries and regions.

However, in the cold spring maize regions of Northeast China (≥10 °C accumulated temperature: 2100–2500 °C, frost-free period: 110–130 days), which are characterized by low accumulated temperature, short frost-free period, and slow straw decomposition, the yield-increasing effect of straw return is often unstable. In some cases, straw return even fails to increase grain yield, leading to the widely reported phenomenon of no yield increase under straw return [10,11,12]. Therefore, optimizing the technical model of straw return and achieving synergistic benefits in the soil–crop system have become key scientific issues for the green transformation of agriculture in the cold regions.

Heilongjiang Province is a major agricultural province and the dominant maize-producing area in Northeast China, with abundant maize straw resources [13]. The local government has issued a series of policies to encourage the resource utilization of straw, promoting the efficient and site-specific utilization of straw to maximize its resource value [14]. Previous studies have shown that maize straw in Heilongjiang is mainly used for livestock feed, domestic fuel, and soil amendment via straw return [15,16]. It has been widely confirmed that straw return can improve soil physical and chemical properties and increase soil nutrient supply, thereby indirectly enhancing crop photosynthetic activity and promoting final yield formation. Nevertheless, most existing studies have focused on the effects of straw return on soil physicochemical properties, while systematic studies linking straw management with maize photosynthetic physiology remain limited [17,18,19].

Long-term straw return and optimized fertilization are widely recognized as effective approaches to improve soil quality and increase crop productivity. However, most studies only focus on the individual effects of straw return or fertilization, and the synergistic mechanism of their coupling on maize photosynthetic characteristics, soil nutrient supply, and yield formation remains unclear. To fill this knowledge gap, a 9-year (2017–2025) long-term fixed-plot field experiment was conducted in the cold spring maize region of Mudanjiang, Heilongjiang. This study systematically compared the effects of straw removal versus straw return, and conventional fertilization versus optimized organic–inorganic fertilization, on maize photosynthetic performance, soil nutrient dynamics, and grain yield. The objectives were to identify the optimal integrated management pattern and clarify the key driving factors of yield. This study provides a scientific and practical basis for optimizing straw return technology and achieving high-yield and sustainable production of spring maize in cold regions.

## 2. Materials and Methods

### 2.1. Site Description

**Figure 1:**
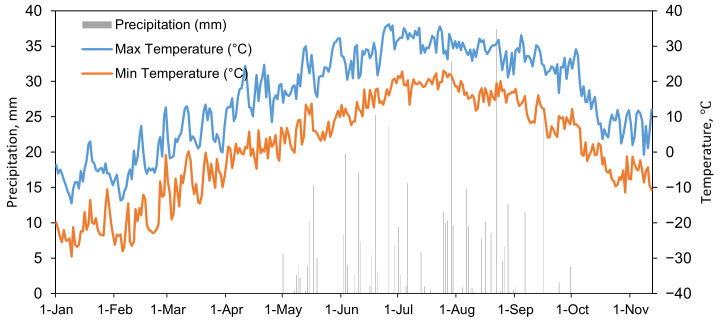
Daily variation characteristics of temperature and precipitation in 2025.

This study was based on a long-term soil quality positioning experiment (i.e., a fixed-site experiment with consistent treatments on the same plots over multiple years) established in 2017 at the Mudanjiang Branch of Heilongjiang Academy of Agricultural Sciences, located in Wenchun Town, Mudanjiang, Heilongjiang, China (44.60° N, 129.58° E). The region has a temperate continental monsoon climate, with a mean annual temperature of 5.0 °C and annual precipitation of 500–600 mm (Figure 1 shows the temperature and precipitation variation for 2025). The soil is classified as meadow soil. Initial soil properties (0–20 cm layer) in 2017 were as follows: bulk density 1.36 g·cm^−3^, soil organic matter 33.28 g·kg^−1^, total nitrogen (TN) 1.09 g·kg^−1^, total phosphorus (TP) 0.95 g·kg^−1^, total potassium (TK) 10.08 g·kg^−1^, alkali-hydrolyzable nitrogen (AN) 74.8 mg·kg^−1^, available phosphorus (AP) 36.76 mg·kg^−1^, available potassium (AK) 172.88 mg·kg^−1^, and pH 7.29.

### 2.2. Experimental Materials

The maize hybrid used was ‘Heyu 236’, with a sowing rate of 25 kg·ha^−1^ and planting density of 68,000 plants·ha^−1^ (65 cm × 22.6 cm). Chemical fertilizers used included urea (N: 46%), diammonium phosphate (N: 18%, P_2_O_5_: 46%), and potassium chloride (K_2_O: 60%). The organic-inorganic compound fertilizer used in the OPT + S treatment contained humic acid (48%), nitrogen (N: 10%), and bio-enzymes.

### 2.3. Experimental Design

A 9-year (2017–2025) field experiment was conducted with five treatments: (1) CK: no fertilizer + straw removal; (2) CF: conventional fertilizer + straw removal; (3) CK + S: no fertilizer + straw return; (4) CF + S: conventional fertilizer + straw return; (5) OPT + S: optimized management with organic-inorganic compound fertilizer + straw return. Each plot covered 156 m^2^ (20 m in length, 12 ridges × 0.65 m). The experiment was arranged in a randomized complete block design with three replications.

Post-harvest straw management was conducted every autumn. After crop harvest, all crop residues were chopped into 5–10 cm pieces using a straw chopper and subsequently incorporated into the soil at a depth of 30–35 cm by moldboard plowing. In the CF and CF + S treatments, conventional fertilizer application involved a basal dressing of urea at 50 kg·ha^−1^, 200 kg·ha^−1^, diammonium phosphate (DAP), and 125 kg·ha^−1^, potassium chloride. An additional 300 kg·ha^−1^, urea was top-dressed at the maize jointing stage. For the OPT + S treatment, organic fertilizer was applied in addition to the conventional basal fertilizer. The application rate of organic fertilizer was optimized as follows: 128.2 kg·ha^−1^ from 2017 to 2021, 37.5 kg·ha^−1^ from 2022 to 2024, and 60 kg·ha^−1^, in 2025. All five treatments were sown manually. Other agronomic practices, including irrigation, weed, and pest control, were kept uniform across all treatments during the entire growing season.

### 2.4. Measurements

#### 2.4.1. Measurement of Maize Photosynthetic Parameters

In 2025, photosynthetic parameters were measured at the jointing, bell-mouth, silking, and filling stages on sunny days from 9:00 to 11:00 under steady light and temperature conditions using a LI-6800XT portable photosynthesis system (LI-COR, Lincoln, NE, USA). Parameters included net photosynthetic rate (Pn), stomatal conductance (Gs), transpiration rate (Tr), and intercellular CO_2_ concentration (Ci). Measurement settings: leaf chamber 2 cm^2^, flow rate 500 μmol·s^−1^, the photosynthetic photon flux density (PPFD) 1200 μmol·m^−2^·s^−1^, leaf temperature was kept consistent with ambient air temperature, and relative humidity was maintained at approximately 55%. Three replicates were measured for each parameter.

#### 2.4.2. Soil Nutrient Analysis

At maize physiological maturity, soil samples were collected from the 0–20 cm layer using a five-point sampling method per plot. Samples from the same plot were mixed, and a portion of the fresh soil was stored at –4 °C for analysis. The remaining soil was air-dried for further analysis. Soil nutrients were determined following standard methods described in Soil Agricultural Chemical Analysis [20]: Total nitrogen (TN) was determined by the Kjeldahl method; available nitrogen (AN) by the alkali hydrolysis diffusion method; P (total/available) were measured by the molybdenum blue colorimetric method; K (total/available) were assessed by flame photometry.

#### 2.4.3. Grain Yield Determination

At maturity, all plants from each plot were harvested manually. Ears were threshed, and grains were air-dried to a constant weight. Grain yield was calculated on a per-hectare basis and adjusted to a standard moisture content of 14%.

### 2.5. Data Processing and Analysis

Statistical analyses were performed using SPSS 26.0 software (IBM Corp., Armonk, NY, USA). Soil nutrients was analyzed by one-way ANOVA and Tukey’s honestly significant difference (HSD) test. A two-way ANOVA was first used to evaluate the main effects of treatment and year and their interaction on maize yield. When the interaction was significant, one-way ANOVA and Tukey’s HSD test were then performed to compare treatments within each year. Repeated-measures ANOVA was applied to analyze the temporal dynamics of photosynthetic parameters across growth stages, where “repeated” indicates multiple measurements on the same experimental units over time. Pearson correlation analysis was used to examine relationships among variables. Figures were generated using Origin 2019 (OriginLab Corp., Northampton, MA, USA) and RStudio 1.2.5033.

## 3. Results

### 3.1. Effects of Straw Return and Fertilization Mode on Photosynthetic Characteristics at Different Growth Stages

#### 3.1.1. Characteristics at the Jointing Stage

**Figure 2:**
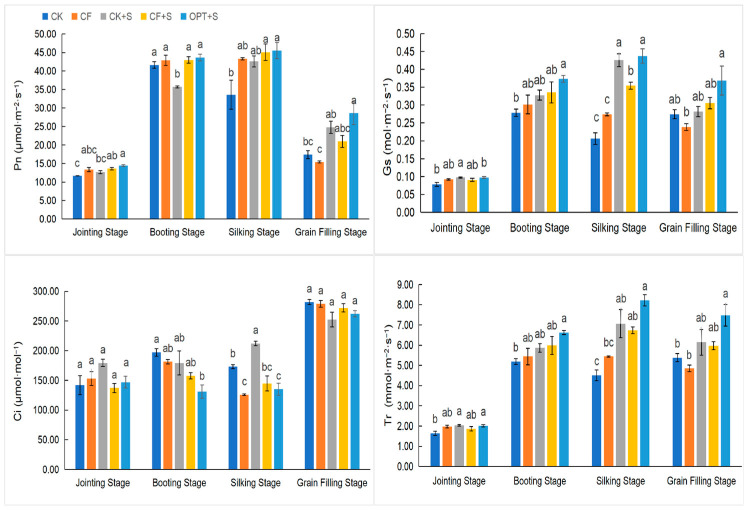
Dynamic changes of photosynthetic physiological characteristics in maize Leaves at different growth stages. Note: Pn, net photosynthetic rate; Gs, stomatal conductance; Ci, intercellular CO_2_ concentration; Tr, transpiration rate. Different lowercase letters indicate significant differences among treatments within the same growth stage (*p* < 0.05). Error bars represent the standard error of the mean.

One-way ANOVA indicated that at the jointing stage, significant differences were detected among treatments in net photosynthetic rate (Pn), transpiration rate (Tr), and stomatal conductance (Gs) (*p* < 0.05), except for intercellular CO_2_ concentration (Ci). (Figure 2). Pn varied the most (F = 8.296, *p* = 0.003), ranging from 11.623 to 14.439 μmol·m^−2^·s^−1^. OPT + S showed the highest Pn, which was 24.2% higher than that of CK and 6.1% higher than that of CF + S. Gs differed significantly (F = 5.440, *p* = 0.014). CK + S increased Gs by 24.6% compared with CK, and OPT + S was significantly higher than CK but statistically similar to CK + S. Tr differed significantly (F = 4.493, *p* = 0.025), with OPT + S and CK + S significantly higher than CK.

#### 3.1.2. Dynamic Changes at the Booting Stage

At the booting stage, a critical transition from vegetative to reproductive growth, photosynthetic parameters showed greater divergence among treatments (Figure 2). The ranking for Pn was OPT + S > CF + S > CF > CK > CK + S. CK + S was significantly lower than the other treatments, while no significant differences were observed among CK, CF, CF + S, and OPT + S. The Ci value of OPT + S was the lowest, significantly lower than CK by 33.5% and lower than CF + S by 16.8%. ANOVA revealed highly significant differences among treatments (F = 5.367, *p* = 0.014). The trend of Gs change was highly consistent with that of Tr, with OPT + S being significantly higher than CK by 34.0% and higher than CK + S by 14.2%.

#### 3.1.3. Critical Role of Photosynthetic Characteristics at the Silking Stage for Yield Formation

The silking stage is a crucial window for photosynthesis determining kernel number and sink capacity, with differences among treatments reaching their peak across the entire growth period (Figure 2). The Tr of the OPT + S treatment was significantly higher than all other treatments (F = 15.818, *p* < 0.001), showing an 82.4% increase over CK. Gs showed the strongest response (F = 44.461, *p* < 0.001), ranging from 0.206 to 0.437 mol·m^−2^·s^−1^, OPT + S increased Gs by 112% compared with CK, and CK + S was statistically similar to OPT + S but superior to other treatments. Ci differed extremely significantly (F = 21.305, *p* < 0.001) and decreased markedly under OPT + S. During this period, Ci decreased significantly while Pn increased significantly, indicating a shift in photosynthetic limitation from stomatal to biochemical factors. The OPT + S treatment effectively broke through this limitation bottleneck by optimizing the carboxylation system.

#### 3.1.4. Senescence and Maintenance Mechanisms of Photosynthetic Characteristics at the Filling Stage

During the filling stage, as leaves senesce, photosynthetic parameters showed a declining trend across all treatments. However, the OPT + S treatment exhibited the strongest ability to maintain photosynthetic capacity (Figure 2). Tr values ranged from 4.853 to 7.488 mmol·m^−2^·s^−1^ and differed significantly among treatments (F = 6.167, *p* = 0.009), with OPT + S being significantly higher than CK and CF. Pn differed extremely significantly (F = 9.099, *p* = 0.002); OPT + S maintained the highest Pn, which was 64.2% and 85.4% higher than CK and CF, respectively. Gs differed significantly (F = 4.889, *p* = 0.019), and OPT + S was significantly higher than CF only. Ci showed no significant divergence among treatments (F = 2.728, *p* = 0.09), though OPT + S remained at a relatively low level.

### 3.2. Interactive Effects of Treatment and Growth Stage on Maize Photosynthetic Characteristics

While the one-way ANOVA revealed stable and consistent differences among treatments at each stage, repeated measures ANOVA further demonstrated that the treatment effects on photosynthetic parameters dynamically evolved with the growth process. Treatment and growth stage exhibited highly significant interactive effects on Pn, Tr, Gs, and Ci (Table 1). The Pn of the OPT + S treatment was consistently and significantly higher than other treatments throughout the growth period, with a decline rate of 37.21%—significantly lower than that of CK (48.20%). The treatment effect on Tr showed a cumulative trend over time. At the jointing stage, the difference between CK + S and OPT + S was not significant; however, this difference expanded to 3.72 mmol·m^−2^·s^−1^ by the silking stage. The Ci of the OPT + S treatment remained at low levels during the jointing, bell-mouthing, and silking stages, with a mean value 21.3% lower than CK. At the silking stage, the Gs of OPT + S peaked at 0.437 mol·m^−2^·s^−1^, 111% higher than CK (*p* < 0.001), and remained at 0.370 during the grain-filling stage, with a decline rate of only 15.3%.

### 3.3. Effects of Straw Return and Fertilization Mode on Soil Nutrients

As shown in Table 2, soil available nutrients responded differently to treatments, with available phosphorus showing the most pronounced variation. The AP content was highest under CF + S (71.72 mg·kg^−1^), significantly exceeding all other treatments and representing a 151.7% increase over CK. OPT + S ranked second, and both CF + S and OPT + S were significantly higher than the treatments without straw return (CK, CF). The AK content showed a gradient of OPT + S > CF + S > CF ≈ CK + S > CK. OPT + S increased AK by 80.7% compared to CK. Treatments with straw return generally increased AK by 15.5% to 80.7% compared to straw removal treatments, indicating a significant effect of straw return on potassium supply. Among total soil nutrients, total potassium content ranged from 8.66 to 9.51 g·kg^−1^, with OPT + S being the highest. All straw return treatments (CK + S, CF + S, OPT + S) had higher total potassium than straw removal treatments (CK, CF).

### 3.4. Effects of Straw Return and Fertilization Regimes on Maize Yield

A two-way analysis of variance (ANOVA) was conducted to systematically examine the main effects of treatment (CK, CK + S, CF, CF + S, OPT + S) and year (2017–2025) on maize grain yield, as well as their interaction. The results indicated that treatment, year, and their interaction all exerted highly significant effects on maize yield (*p* < 0.001). The overall model explained 90.8% of the total variation in yield (R^2^ = 0.908, adjusted R^2^ = 0.863), demonstrating a good fit. The effect of treatment (F = 156.71) was significantly stronger than that of year (F = 5.37), indicating that fertilization and straw management practices were the core factors regulating yield differences. The significant treatment × year interaction (F = 6.86, *p* < 0.001) suggested that the yield performance of different treatments exhibited obvious interannual heterogeneity (Table 3).

**Figure 3:**
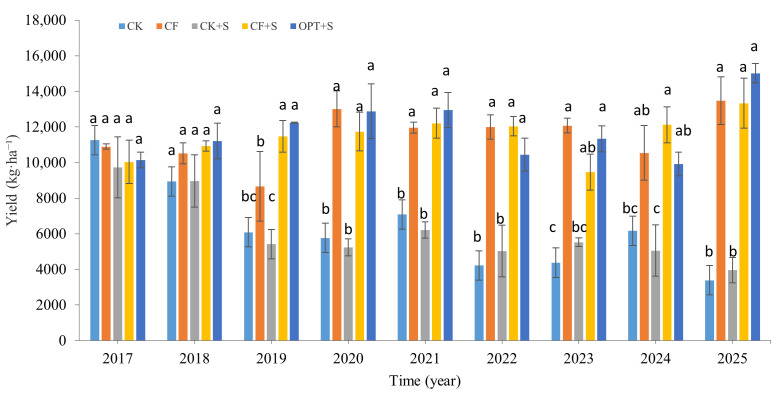
Interannual dynamics of maize grain yield under different fertilization and straw return treatments from 2017 to 2025. Data are presented as means ± standard error. Different lowercase letters indicate significant differences among treatments in the same year (*p* < 0.05).

When the interaction was significant (Figure 3), One-way ANOVA was conducted to test the effect of fertilization treatments (CK, CK + S, CF + S, OPT + S, CF) on maize yield, followed by Levene’s test for homogeneity of variances and Tukey’s HSD post-hoc multiple comparisons. The results showed that the yields of CK and CK + S treatments generally decreased over the years, with 11,261.83 kg·ha^−1^ and 9726.29 kg·ha^−1^ in 2017, respectively, and decreased to 3386.65 kg·ha^−1^ and 3952.96 kg·ha^−1^ in 2025, representing reductions of 69.93% and 59.36%. The yield decline in CK + S was lower than that in CK, and the difference between the two treatments was not significant in most years. In contrast, the yields of CF + S and OPT + S treatments showed an overall upward trend, though slight decreases occurred during 2022–2024. Over the nine-year period, CF + S increased yield by 32.83%, and OPT + S increased yield by 4871.11 kg·ha^−1^ compared with the initial year, corresponding to an increase of 48.01%. Moreover, the yield differences among treatments gradually expanded with the extension of the experimental years. Post-hoc comparisons indicated that in 2025, the yield of OPT + S reached 15,016.11 kg·ha^−1^, which was significantly higher than the control treatments (*p* < 0.001), representing yield increases of 270.5% compared with CK and 9.9% compared with CF + S, respectively. The yields of CF and CF + S treatments ranked just below that of OPT + S, and no significant differences were observed among these three treatments.

### 3.5. Correlation and Integrated Effects Among Maize Photosynthesis, Soil Properties, and Yield

**Figure 4:**
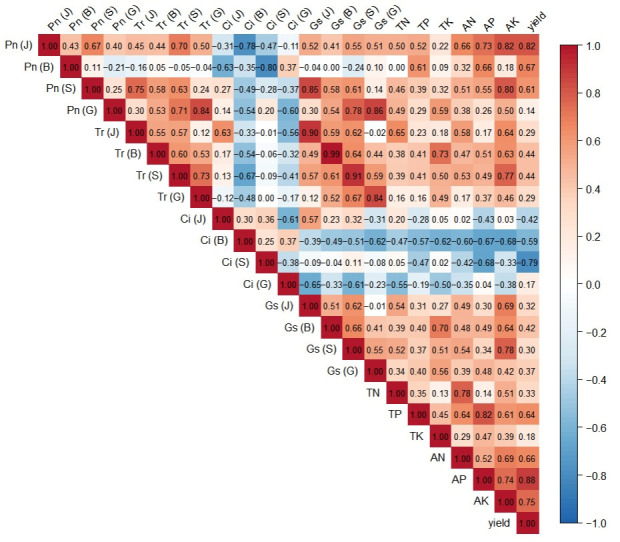
Interparameter correlation matrix for maize photosynthesis, soil properties, and yield. Abbreviations: J, jointing stage; B, booting stage; S, silking stage; G, grain filling stage. Values in parentheses indicate the growth stage.

Pearson correlation analysis was used to reveal the relationships among yield, photosynthetic physiology, and soil nutrients (Figure 4). Correlation analysis only indicates the direction and strength of linear relationships and does not represent the importance or contribution of variables. Using 2025 yield data, correlation analysis showed that yield was significantly positively correlated with available phosphorus (R = 0.876, *p* < 0.001), followed by Pn at the jointing stage (R = 0.821, *p* < 0.001) and available potassium (R = 0.747, *p* < 0.001).

The correlation between photosynthetic physiology and yield varied across growth stages. The correlation of Pn at the jointing stage on yield was significantly higher than that of Pn at the silking and filling stages. Ci at the silking stage showed a significant negative correlation with yield.

For soil nutrients, available phosphorus and available potassium were significantly correlated with yield (R = 0.876 and 0.747, respectively, *p* < 0.001). Pn at the jointing stage was highly coupled with available potassium (R = 0.823) and also significantly correlated with available phosphorus (R = 0.727). Ci at the silking stage showed significant negative correlations with available phosphorus and potassium (R = −0.680 and −0.333, respectively). Stepwise regression and standardized coefficients further confirmed that available phosphorus and Pn at the jointing stage were the key driving factors for yield (R^2^ = 0.84, *p* < 0.001).

In summary, the coordination between early-stage photosynthetic capacity and soil available phosphorus and potassium supply was closely associated with yield formation in cold-region spring maize.

**Figure 5:**
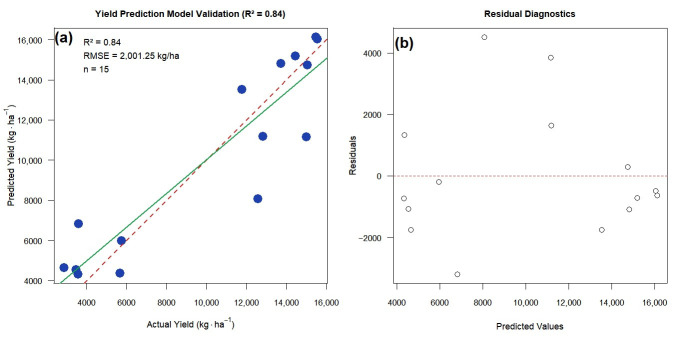
Yield prediction model and residual diagnostic plot.

Stepwise regression was used to construct a yield prediction model, which automatically retained available phosphorus (AP) and photosynthetic rate at the jointing stage (PnJ). The regression equation was: Yield = −21,028.21 + 157.54 × AP + 1804.81 × PnJ (R^2^ = 0.840, n = 15, *p* < 0.05). Standardized coefficients indicated that the contribution of available phosphorus (Beta = 0.59) was greater than that of photosynthetic rate (Beta = 0.39). The validity of the stepwise regression model was verified using a scatter plot of predicted vs. actual values (Figure 5a). The points were randomly scattered around the 1:1 line, with R^2^ = 0.84 and RMSE = 2001.25 kg·ha^−1^, indicating a good model fit and reliable predictive accuracy. This confirmed the robustness of the dual-factor driving mechanism involving available phosphorus and jointing stage photosynthesis. The residual diagnostic plot (Figure 5b) showed that residuals were randomly distributed around the zero axis with no systematic bias, indicating that the regression model met the assumptions of linearity, normality and homoscedasticity.

## 4. Discussion

### 4.1. Physiological Mechanisms Underlying Photosynthetic Enhancement Driven by the Synergy of Straw Return and Fertilization

Leaves are the key functional organs for crop photosynthesis, serving as the primary sites for light capture and gas exchange, with over 90% of biomass accumulation derived from photosynthesis [21,22,23]. This study demonstrates that the synergistic treatment of straw return with organic-inorganic compound fertilizer (OPT + S) exhibited superior photosynthetic performance throughout the entire growth period, especially at the silking and filling stages (highest Pn and Gs, lower Ci). This result surpassed the treatment with chemical fertilizer alone (CF + S), revealing the limitations of single straw return or pure chemical fertilizer application. From a physiological perspective, the OPT + S treatment showed a significantly lower photosynthetic decline rate during the filling stage (37.3%) compared to CK (48.2%), indicating stronger photosynthetic sustainability. This is likely attributed to the organic components (e.g., humic acid, bio-enzymes) in the organic-inorganic compound fertilizer, which may act as biostimulants that directly or indirectly regulate the balance of endogenous plant hormones, delay the expression of genes related to leaf senescence, and help maintain chloroplast structure and Photosystem II (PSII) activity during the grain-filling period, aligning with trends reported by Gao and co-authors [24,25,26,27]. Furthermore, organic materials release nitrogen, phosphorus, potassium, and trace elements continuously and steadily through mineralization processes during the middle and late growth stages, avoiding potential nutrient depletion that can occur with pure chemical fertilizers and providing sustained support for leaf function. In cold regions, however, straw return may also cause potential problems such as nitrogen immobilization, which will be focused on and optimized in future research.

### 4.2. Driving Role of Soil Nutrient Availability in Yield Formation

This study highlights that in the cold spring maize system, soil available phosphorus (AP) and available potassium (AK) are the core soil factors linking crop photosynthesis and final yield, with their correlation to yield being significantly stronger than that of total nutrients. This underscores that under the ecological constraints of low temperatures and a short growing season, the bioavailability and supply intensity of soil nutrients are more critical than their total reserves. Both OPT + S and CF + S treatments significantly increased soil AP content, with CF + S being the highest. This primarily results from the direct input of chemical phosphorus fertilizer and the potential activation of fixed soil phosphorus by straw return through altering soil pH and increasing organic acid secretion. The OPT + S treatment achieved the highest AK content, likely benefiting from the chelating and slow-release effects of the organic components in the compound fertilizer and the increased potassium input from additional organic–inorganic fertilizer reducing potassium leaching, which echoes the findings of Gong and co-authors [28,29]. Notably, despite CF + S having the highest AP, its yield was significantly lower than OPT + S. This indicates that merely increasing the concentration of a single available nutrient in the soil is insufficient to maximize system productivity. The advantage of OPT + S lies in the coordination and synchrony of nutrient supply. The organic components likely optimize the release dynamics of nitrogen, phosphorus, and potassium by promoting soil microbial activity, making nutrient availability better match the demands of maize at different growth stages, thereby achieving a more efficient transformation from “high soil phosphorus” to “high crop yield” [30].

Since the OPT treatment (optimized fertilization without straw return) was not set in this study, the independent effects of straw return and fertilization could not be distinguished quantitatively. Future studies will include the OPT treatment to further distinguish the separate and synergistic effects of fertilization and straw return.

### 4.3. Regulation of the Photosynthesis-Soil-Yield Coupling Pathway by Straw Return and Fertilization Mode

Many scholars have suggested that photosynthetic rate at specific growth stages are positively correlated with yield [31,32]. Feng and co-authors pointed out a close relationship between photosynthetic rate at the silking stage and yield in maize [33]. However, the stepwise regression analysis in this study identified the core pathway as “Pn at jointing stage → Soil Available Phosphorus → Yield”. This discrepancy with previous studies may stem from the unique conditions of cold, short-season regions. Here, the entire crop growth cycle is compressed, making early biomass accumulation and organ establishment particularly critical. The jointing stage is a period of vigorous vegetative growth, rapid root expansion, and initiation of ear differentiation. High photosynthetic efficiency at this stage means more photosynthetic products can be allocated to the roots, promoting the development of a robust and active root system. The OPT + S treatment increased jointing stage Pn by 24.2% compared to CK, directly promoting the translocation of photosynthetic products to roots and driving soil phosphorus activation. High levels of available phosphorus in the soil (contributed jointly by fertilization and straw return) then ensure the roots have “phosphorus to absorb.” The absorbed phosphorus further supports metabolic activity in leaves during the middle and late stages and grain filling, forming a positive feedback loop: “early photosynthetic foundation → robust root system construction → efficient phosphorus uptake → mid-to-late stage photosynthetic and yield support.”

## 5. Conclusions

Based on the 9-year experiment (2017–2025) and systematic measurements in 2025, the OPT + S treatment is the optimal practice for green and high-yield spring maize production in cold regions. This treatment maintained a high net photosynthetic rate during the whole growth period, and the photosynthetic decline rate during the filling stage was only 37.21%, which was significantly lower than that of CK (48.20%). Such a benefit helped delay leaf senescence and promote grain filling and yield formation. Meanwhile, OPT + S significantly increased soil available phosphorus and available potassium contents, and improved maize yield through the synergistic interaction between soil nutrient supply and leaf photosynthesis. In 2025, the grain yield under OPT + S reached 15,016.11 kg·ha^−1^. Comprehensive analysis indicated that net photosynthetic rate at the jointing stage and soil available phosphorus content were the core driving factors regulating yield formation of spring maize in cold regions. As the treatment of optimized fertilization without straw return (OPT) was not included in this study, the independent effects of fertilization and straw return could not be accurately distinguished. Future studies should add the OPT treatment to further clarify the synergistic mechanism of optimized fertilization and straw return, and pay attention to potential negative effects such as nitrogen immobilization caused by straw return.

## Figures and Tables

**Table 1:** Results of between-subject effects test for the effects of different treatments and growth stages on maize photosynthetic characteristics.

	Pn (μmol·m^−2^·s^−1^)			Gs (mol·m^−2^·s^−1^)			Tr (mmol·m^−2^·s^−1^)			Ci (μmol·mol^−1^)		
	df	F	*p*	df	F	*p*	df	F	*p*	df	F	*p*
Treatment	4	11.080	<0.001	4	235.259	<0.001	4	23.615	<0.001	4	10.247	<0.001
Growth Stage	3	436.582	<0.001	3	27.448	<0.001	3	226.586	<0.001	3	173.499	<0.001
Treatment × Stage	12	5.176	<0.001	12	6.637	<0.001	12	3.918	0.001	12	5.285	<0.001

Note: df, degrees of freedom; F, F-value; *p*, significance level (*p* < 0.001 indicates highly significant difference).

**Table 2:** Effects of different treatments on soil nutrients at maturity under long-term positioning conditions.

Treatment	TN (g·kg^−1^)	TP (g·kg^−1^)	TK (g·kg^−1^)	AN (mg·kg^−1^)	AP (mg·kg^−1^)	AK (mg·kg^−1^)
CK	0.95 ± 0.06 a	0.86 ± 0.01 b	8.99 ± 0.03 ab	82.50 ± 0.66 b	28.49 ± 0.42 d	213.96 ± 2.57 c
CF	1.09 ± 0.07 a	0.91 ± 0.03 ab	8.66 ± 0.32 b	95.73 ± 0.87 ab	45.17 ± 0.86 c	334.43 ± 8.76 b
CK + S	1.10 ± 0.04 a	0.86 ± 0.01 b	9.06 ± 0.36 ab	90.15 ± 2.28 ab	25.42 ± 0.58 e	334.04 ± 3.05 b
CF + S	1.01 ± 0.08 a	1.05 ± 0.07 a	9.23 ± 0.09 ab	91.60 ± 0.65 ab	71.72 ± 1.69 a	378.32 ± 10.92 a
OPT + S	1.13 ± 0.16 a	1.01 ± 0.13 ab	9.51 ± 0.10 a	100.12 ± 11.72 a	65.30 ± 1.62 b	386.50 ± 3.12 a

Note: Values are mean ± standard deviation (SD). Different lowercase letters within a column indicate significant differences at *p* < 0.05 according to Tukey’s HSD test. TN: total nitrogen; TP: total phosphorus; TK: total potassium; AN: alkali-hydrolyzable nitrogen; AP: available phosphorus; AK: available potassium.

**Table 3:** Two-way ANOVA results for maize grain yield as affected by treatment and year.

	Type III SS	df	Mean Square	F-Value	*p*-Value
Treatment	924,389,485.4	4	231,097,371.4	156.709	<0.001
Year	63,332,290.90	8	7,916,536.363	5.368	<0.001
Treatment × Year	323,496,574.4	32	10,109,267.95	6.855	<0.001

R^2^ = 0.908 (Adjusted R^2^ = 0.863).

## Data Availability

The original contributions presented in this study are included in the article; further inquiries can be directed to the corresponding author.
